# Toward a Brain-Inspired System: Deep Recurrent Reinforcement Learning for a Simulated Self-Driving Agent

**DOI:** 10.3389/fnbot.2019.00040

**Published:** 2019-06-28

**Authors:** Jieneng Chen, Jingye Chen, Ruiming Zhang, Xiaobin Hu

**Affiliations:** ^1^Department of Computer Science, College of Electronics and Information Engineering, Tongji University, Shanghai, China; ^2^School of Computer Science, Fudan University, Shanghai, China; ^3^Department of Computer Science, Technical University of Munich, Munich, Germany

**Keywords:** self-driving agent, brain-inspired learning, reinforcement learning, end-to-end architecture, recurrence

## Abstract

An effective way to achieve intelligence is to simulate various intelligent behaviors in the human brain. In recent years, bio-inspired learning methods have emerged, and they are different from the classical mathematical programming principle. From the perspective of brain inspiration, reinforcement learning has gained additional interest in solving decision-making tasks as increasing neuroscientific research demonstrates that significant links exist between reinforcement learning and specific neural substrates. Because of the tremendous research that focuses on human brains and reinforcement learning, scientists have investigated how robots can autonomously tackle complex tasks in the form of making a self-driving agent control in a human-like way. In this study, we propose an end-to-end architecture using novel deep-Q-network architecture in conjunction with a recurrence to resolve the problem in the field of simulated self-driving. The main contribution of this study is that we trained the driving agent using a brain-inspired trial-and-error technique, which was in line with the real world situation. Besides, there are three innovations in the proposed learning network: raw screen outputs are the only information which the driving agent can rely on, a weighted layer that enhances the differences of the lengthy episode, and a modified replay mechanism that overcomes the problem of sparsity and accelerates learning. The proposed network was trained and tested under a third-party OpenAI Gym environment. After training for several episodes, the resulting driving agent performed advanced behaviors in the given scene. We hope that in the future, the proposed brain-inspired learning system would inspire practicable self-driving control solutions.

## 1. Introduction

Recently, research in brain science has gradually received the public's attention. Given the rapid progress in brain imaging technologies and in molecular and cell biology, much progress has been made in understanding the brain at the macroscopic and microscopic levels. Currently, the human brain is the only truly general intelligent system that can cope with different cognitive functions with extremely low energy consumption. Learning from the information processing mechanisms of the brain is clearly the key to building stronger and more efficient machine intelligence (Poo et al., 2016). In recent years, some bio-inspired intelligent methods have emerged (Marblestone et al., 2016; Gershman and Daw, 2017; Hassabis et al., 2017; Botvinick et al., 2019), and they are clearly different from the classical mathematical programming principle. Bio-inspired intelligence has the advantages of strong robustness and an efficient, well distributed computing mechanism. It is also easy to combine with other methods.

The mammalian brain has multiple learning subsystems. Niv (2009) categorized major learning components into four classes: the neocortex, the hippocampal formation (explicit memory storage system), the cerebellum (adaptive control system), and the basal ganglia (reinforcement learning). Among these learning components, reinforcement learning is particularly attractive to research. Nowadays, converging evidence links reinforcement learning to specific neural substrates, thus assigning them to precise computational roles. Most notably, much evidence suggests that the neuromodulator known as dopamine provides basal ganglia target structures with phasic signals that convey a reward prediction error which can influence learning and action selection, particularly in stimulus-driven habitual instrumental behaviors (Rivest et al., 2005). Hence, many efforts have been made to investigate the capability of bio-inspired reinforcement learning by applying them to artificial intelligence-related tasks (Peters and Schaal, 2008; Mnih et al., 2015; Zhu et al., 2017; Gu et al., 2017).

In recent years, deep reinforcement learning has contributed to many of the spectacular success stories of artificial intelligence (Kober et al., 2013; Henderson et al., 2018). After the initial success of the deep Q network (DQN) (Mnih et al., 2013), a variety of improved models have been published successively. Later on and based on the former discoveries, Mnih et al. (2015) proposed the Nature DQN in 2015 and introduced the replay memory mechanism to break the strong correlations between the samples. Mnih et al. (2016) proposed a deep reinforcement learning approach, in which the parameters of the deep network are updated by multiple asynchronous copies of the agent in the environment. Van Hasselt et al. (2016) suggested the Double DQN to eliminate overestimation; they added a target Q network independent from the current Q network. It was shown to apply to large-scale function approximation (Van Hasselt et al., 2016). Wolf et al. (2017) applied a deep Q network to a driving scenario in a physics simulation based track. Newer techniques included deep deterministic policy gradients and mapping an observation directly to action, both of which could operate over continuous action spaces (Lillicrap et al., 2016). Schaul et al. (2016) suggested prioritized replay, adding priority to replay memory to relieve the sparse reward and slowly converge on the problem (Schaul et al., 2016). Reviewed in Hassabis et al. (2017), experience replay was inspired by theories that seek to understand how the memory system in the mammalian brain might interact, and thus has biological plausibility. In the case of partially observable states, the recurrent neural network (RNN) and long short-term memory (LSTM) have been proven to be effective in processing sequence data (Hochreiter and Schmidhuber, 1997). Hausknecht and Stone (2015) replaced the last fully connected layer in the network with an LSTM layer. They integrated information through time and replicated DQN's performance on standard Atari games and partially observed equivalents featuring flickering game screens. Also, Foerster et al. (2016) proposed to use multi-agent to describe a state distributively. A recent work (Kahn et al., 2018) adopted double Q learning with recurrency and computation graphs to tackle a robotics navigation task. Nevertheless, some previous studies, such as those with Atari games, focused on the simple environment and action space. Moreover, the previous studies do not provide comprehensive comparisons with supervised learning in a specific scenario. Because of these limitations, there is an urgent need to further improve the capability of deep reinforcement learning in a more challenging and complex scenario such as the simulated driving control problem.

To clarify the biological plausibility, Lake et al. (2017) state that there is indeed substantial evidence that the brain uses similar model-free RL learning algorithms in simple associative learning or discrimination learning tasks. In particular, the phasic firing of midbrain dopaminergic neurons is qualitatively and quantitatively consistent with the reward prediction error that drives updating of value estimates. In the process of reinforcement learning, the agent's attempt in each state was like the regulation process of dopamine in the brain (Dolan and Dayan, 2013). To the best of our knowledge, there is rare work studying the behavior difference between supervised learning and RL in a specific scenario. In the kart driving case in this work, the proposed learned agent shows stronger biological plausible learning capability than the supervised learned agent, in respect to dealing with specific situations and its adaptability.

One supervised learning-based study looked at the simulated self-driving game (Ho et al., 2017). However, three problems existed in their implementation. First, they created a handcrafted dataset. Obviously, one can never create this ideal benchmark dataset that includes all the bad situations encountered by the driving agent during training. At best, one can include the best behavior that the driving agent should implement in each step. The driving agent was reported to perform well when it had a good position in the driveway. However, the behavior deteriorated rapidly when the driving agent deviated from the driveway. Such behaviors indicated the dissimilar distribution and instability even though correctional measures were taken on the dataset. Second, they trained and supervised their network in a supervised way. As there are many possible scenarios, manually tackling all possible cases using supervised learning methods will likely yield a more simplistic policy (Shalev-Shwartz et al., 2016). Third, their experiments were built on ideal conditions; for example, they assumed that the brakes were ignored. In our experiments, we take the brakes into consideration. Moreover, to support autonomous capabilities, a robotic driven agent should adopt human driving negotiation skills when braking, taking left and right turns, and pushing ahead in unstructured roadways. It comes naturally that a trial-and-error way of study is more suitable for this simulated self-driving game. Hence, the bio-inspired reinforcement learning method in the study is a more suitable way for the driving agent to learn how to make decisions.

In our study, we proposed a deep recurrent reinforcement learning network to solve simulated self-driving problems. Rather than creating a handcrafted dataset and training in a supervised way, we adopted a bio-inspired trail-and-error technique for the driving agent to learn how to make decisions. Furthermore, this paper provides three innovations. First, intermediate game parameters were completely abandoned, and the driving agent relied on only raw screen outputs. Second, a weighting layer was introduced in the network architecture to strengthen the intermediate effect. Third, a simple but effective experience recall mechanism was applied to deal with the sparse and lengthy episode.

The rest of this study is organized as follows: section 2 describes deep Q-learning, recurrent reinforcement learning, network architecture, and implementation details. Section 3 verifies experimental results. The conclusion of this study is drawn in section 4.

## 2. Methodology

Deep Q-learning is used to help AI agents operate in environments with discrete actions spaces. Based on the knowledge of Deep Q-learning, we proposed a modified DRQN model in order to infer the full state in partially observable environments.

### 2.1. Deep Q-Learning

Reinforcement learning manages learning policies for an agent interacting in an unknown environment. In each step, an agent observes the current states of the environment, makes decisions according to a policy π, and observes a reward signal *r*_*t*_ (Lample and Chaplot, 2017). Given the current states and a set of available actions, the main aim of the DQN is to approximate the maximum sum of discounted rewards. According to the Bellman equation, it gives the approximating form of Q-values by combining the reward obtained with the current state-action pair and the highest Q-value at the next state *s*_*t*+1_, and the best action *a′*:

(1) $$Q\left(s_{t},a_{t}\right)\leftarrow r_{t}+\gamma *\underset{a^{\prime }}{\text{arg max }}Q\left(s_{t+1},a\prime \right)$$

We often use the form involving an iterative process:

(2) $$Q\left(s_{t},a_{t}\right)\leftarrow Q\left(s_{t},a_{t}\right)+\alpha \left(r_{t}+\gamma *\underset{a^{\prime }}{\text{arg max }}Q\left(s_{t+1},a^{\prime }\right)-Q\left(s_{t},a_{t}\right)\right)$$

In the assignments above, α stands for the learning rate and γ stands for the discounted factor.

The agent chooses the action following a ε-greedy exploration policy. The value of ε ranges from 0.0 to 1.0. In order to encourage the agent to explore the environment, the ε was set to 1.0 at first. During the training process, the value decreased gradually as the experience accumulated. Then, the agent could use experience to complete the task.

When we sample a sequence (*s*_*t*_, *a*_*t*_, *r*_*t*_, *s*_*t*+1_) from the replay memory unit, the target value *y*_*t*_ is calculated as:

(3) $$y_{t}=\{\begin{array}{ll} r_{t} & \text{ for terminal }s_{t+1}\\ r_{t}+\gamma \arg\max_{a^{\prime }}Q\left(s_{t+1},a^{\prime }|\theta \right) & \text{ for non-terminal }s_{t+1} \end{array}$$

The network was trained to approximate the expected Q-value, which led to the loss function, with parameters θ in the model:

(4) $$\begin{array}{l} \mathrm{Loss}\left(\theta \right)=\sum {\left(y_{t}-Q\left(s_{t},a_{t}|\theta \right)\right)}^{2} \end{array}$$

### 2.2. Recurrent Reinforcement Learning

**Figure 1:**
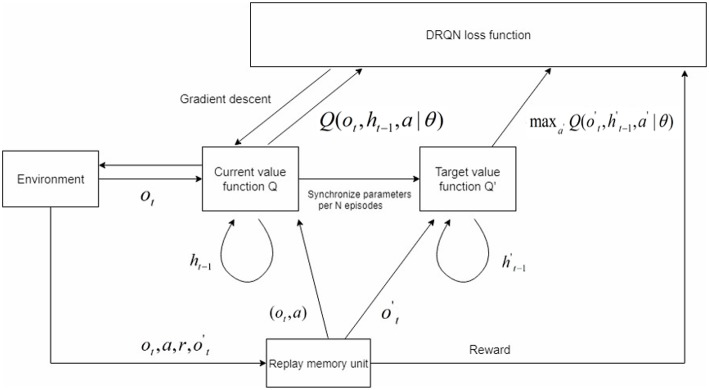
The modified DRQN model. The value function was divided into two categories: the current value function *Q* and target value function *Q′*. The parameters in *Q* were assigned to *Q*′ per N episodes. The state contained two elements: *o*_*t*_ gained from the current environment and *h*_*t*−1_ gained from former information. The agent performed action *a* using a specific policy, and the sequence (*o*_*t*_, *a, r, o*_*t*_′) was stored in the replay memory unit. We used a prioritized experience replay memory unit here. During training, the sequence was randomly chosen from the replay memory unit. We trained the network using gradient descent to make the current value function *Q* approach *Q′* given a specific sequence. The loss function was shown in Equation (4).

**Figure 2:**
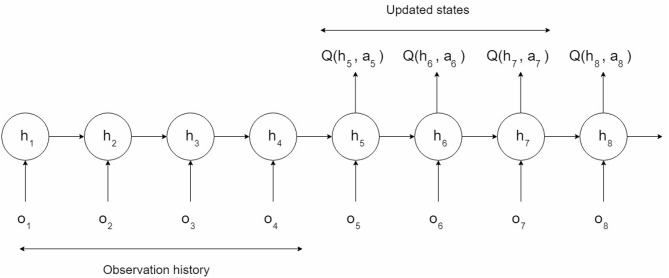
Sequence updates in the recurrent network. Only the scores of the actions taken in states 5, 6, and 7 will be updated. The first four states provide a more accurate hidden state to the LSTM, while the last state provides a target for state 7.

For some special games which are three-dimensional and partially observable, the DQN lacks the ability to solve the problem. In partially observable environments, the agent only receives an observation *o*_*t*_ of the current environment, which is usually insufficient to infer the full state of the system. The real state *s*_*t*_ is the combination of the current observation *o*_*t*_ and an unfixed length of history states. Hence, we adopted the DRQN model on top of the DQN to deal with such conditions (see Figure 1). The last fully connected layer was replaced by the LSTM in the DRQN model in order to record former information. Figure 2 shows the sequential updates in the recurrent network. When updating the DRQN model, a sequence S was randomly sampled from the replay memory unit, and the beginning time step *t* was also randomly chosen according to the maximum length *l*. Then the cut sequence *S*_*t, t*+1, …, *l*−1, *l*_ was sent to the DRQN model. An additional input *h*_*t*−1_ standing for the previous information was added to the recurrent model. At the zero time step, *h*_*t*−1_ was set to zero. The output of the LSTM *z*(*o*_*t*_, *h*_*t*−1_), which combined the current observation *o*_*t*_ and the history information *h*_*t*−1_, was used to approximate the Q-value *Q*(*o*_*t*_, *h*_*t*−1_, *a*_*t*_). The history information was updated and passed through the hidden state to the network in the next time step:

(5) $$\begin{array}{l} h_{t}=LSTM\left(h_{t-1},o_{t}\right) \end{array}$$

### 2.3. Network Architecture

**Figure 3:**
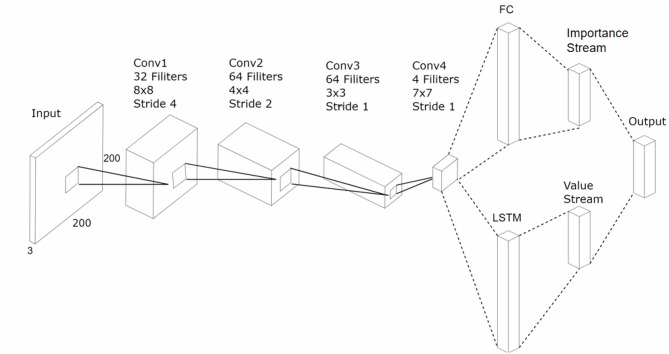
An illustration of the architecture of our model. The input image is assigned to four convolution layers. The output of the convolution layers is split into two streams. The first stream (bottom) flattens the output and feeds it to an LSTM. The second one (top) flattens the output and feeds it to a fully connected layer. Then, we obtained an importance stream and value stream individually and multiplied them as the output. We stored the information in prioritized experience memory unit. As was shown in Figure 1, the network was trained using the DRQN loss function.

In the beginning, we used the baseline DRQN model (Lample and Chaplot, 2017) to make an agent perform self-driving. However, we obtained unsatisfied results with the same model. Hence, we have made three improvements in our modified model to make things work better. The whole architecture is shown in Figure 3. First, the network was built on top of the NVIDIA's autopilot model (Krizhevsky et al., 2012). To reduce overfitting, the original model was modified by adding several batch normalization layers. We used a four-layer stronger CNN for feature extraction. The input size was resized to 320*240. The first convolutional layer contained 32 kernels, with a size size of 8*8 and a stride of 4. The second convolutional layer contained 64 kernels, with a size of 4*4 and a stride of 4. The third layer contained 128 kernels, with a size of 3*3 and a stride of 1. The last convolutional layer contained 256 kernels with a size of 7*7 and a stride of 1. Relu was used as the activated function in the network, and the sizes of the pooling layers were all 2*2. Second, we abandoned the fully connected layers before the LSTM layer in the original DRQN model and fed the LSTM directly with the high-level feature. The number of units in LSTM was set to 900. Third, the subsequent structure was divided into two groups for different purposes. One was mapped to the set of possible actions, and the other was a set of scalar values. The final action value function was value function was acquired using both of them. We will introduce their functions respectively.

We used two collateral layers rather than a single DRQN network to approximate the value function. The auxiliary layer also had a five-dimensional output as the action space layer. The original target was to balance the impact of the current and history. The agent could make a suitable action with a fully observing perspective. Nevertheless, we wanted to focus on the precise instantaneous changes of the current scene. The output of the auxiliary layer was mapped to [0, 1.0] using the *softmax* function, thus suggesting the correction for the raw approximations using the DRQN model. Because of this intervention, the network would not only learn the best action for a state but also understand the significance of taking actions. If an agent drove in a straight line and had an obstacle far ahead, an original DRQN model could learn that it was time to make the driving agent move a bit to avoid hitting the obstacle. The modified model would also have insight into when it was the best time to move, with the knowledge that the danger of the obstacle increases as it gets closer. *V*(*s, a*) was used to represent the original output of the DRQN, and *I*(*s, a*) was used to represent the importance provided by the auxiliary layer. We used the formula to express the final strengthen of the Q-value (see Figure 3):

(6) $$\begin{array}{l} Q\left(s,a\right)=V{\left(s,a\right)}^{T}*I\left(s,a\right) \end{array}$$

The result was stored in the prioritized experience memory unit. During training, the sequence in the memory unit was removed, and we used Equation (4) to calculate the loss.

### 2.4. Implementation Details

Reinforcement learning consists of two basic concepts: action and reward. Action is what an agent can do in each state. Given that the screen is the input, a robot can take steps within a certain distance. An agent can take finite actions. When a robot takes an action in a state, it receives a reward. Here, the term reward is an abstract concept that describes the feedback from the environment. A reward can be positive or negative. When the reward is positive, it corresponds to our normal meaning of reward. However, when the reward is negative, it corresponds to what we usually call punishment. We also describe the training details such as the hyperparameters, input size selection, frameskip, and prioritized replay.

#### 2.4.1. Action Space

The agent could perform five actions, including Left, Right, Straight, Brake, and Backwards. The range of the joystick reflects the numerical value of the speed control, which is mapped into a region of –80 to 80. The speed section was discretized into a set of [0, 20, 40, 80] for speed control. We considered that only the turning control had a high requirement for precision to keep the model simple. Another three actions, including forward flag, backward flag and brake, were represented by 1/0 flag. Thus, we used 5-dimensional vectors to represent each action as shown below:

$$\begin{array}{cc} actions & =[40,0,1,0,0],\;left\\ & =[-40,0,1,0,0],\;right\\ & =[0,-80,0,1,0],\;go\;backwards\\ & =[0,0,1,0,0],\;go\;straight\\ & =[0,0,0,0,1],\;brake \end{array}$$

The meaning of each dimension in the vector represented forward speed, backward speed, backward flag, forward flag, and brake. For forward speed, a positive value indicates to the left and a negative value indicates to the right. When the backward flag was set to 1, the agent would move backwards at a specific speed.

#### 2.4.2. Reward

Mnih et al. (2015) states that end-to-end human-level RL control draws on neurobiological evidence that reward signals during perceptual learning may influence the characteristics of representations within the primate visual cortex. The AI trained in a supervised way would only respond to visual information. If the kart picks the wrong direction, it would likely drive straight since the scenes of the correct and wrong direction are mostly the same. In other words, the supervised learned agent does not understand risky situations that are likely to lead into error states during real-time play. In contrast, an agent receiving reward and punishment signals can avoid the noted situation efficiently. Under most circumstances, the driving agent cannot explore a path with big rewards initially. The driving agent often gets stuck somewhere in halfway through and waits for the time to elapse before resetting. We have to make the rewards of these cases variant in order to make these experiences meaningful. Hence, we set a series of checkpoints along the track. A periodical reward was given to the driving agent when each checkpoint was reached. The closer the distance between the checkpoint and the destination, the bigger the phased reward was given. We have established a more precise reward system to increase density, such as giving the driving agent a slight punishment when it moves backwards. At each step, the agent will also get a little punishment. The detailed component of the reward will be introduced in section 2.4.4.

#### 2.4.3. Prioritized Replay

Hassabis et al. (2017) states that experience replay was directly inspired by theories that seek to understand how the memory systems in the mammalian brain might interact. According to the biological plausibility mentioned in Hassabis et al. (2017) and Schaul et al. (2016), we modified the original replay mechanism. In most cases, there was little replay memory with high rewards, which would be time-consuming with a huge replay table and many sparse rewards. As prioritized replay was a method that can make learning from experience replay more efficient, we simply chose the important experiences in proportion to their rewards and stored them into the replay memory. That way, memories with high rewards would have a greater opportunity to be recalled.

#### 2.4.4. Hyperparameters

The original screen outputs were three channel RGB images. They were first transformed into gray-scale images and then fed to the network to train. The network was trained using the RMSProp algorithm. The size of minibatch was set to 40. The size of replay memory was set to contain 10,000 recent frames. The learning rate α was set to 1.0 in the beginning and followed a linear degradation and finally was fixed at 0.1. The exploration rate ε was set to 0 when we evaluated the model. When the agent finished the game, it would get 1,000 scores as reward. Whenever it crossed each lap, it would get 100 scores. The agent would get 0.5 scores as it got to a checkpoint. If the agent moved backwards, it would get –0.5 scores as punishment. At each time step, it would get –0.1 scores as punishment because we wished the agent to finish the game as quickly as possible.

#### 2.4.5. Other Details

To accelerate training and save running memory, the original 640*480 screen resolution was resized to 160*120 in the beginning. After several hours of trials, the driving agent still got stuck in most cases and could not complete one lap. The resulting rewards oscillated for not finishing the game in the limited number of steps, thus indicating the resolution was too low for the model to recognize. To enrich the visual information and address the above problems, the input size was set to 320*240. We also attempted to reduce the punishments to encourage positive rewards. After the observation of the same length of time, the distribution of the resulting rewards became steady and started to turn positive over the baseline. Hence, the input size of the resolution was finally set to 320*240 in the experiment in spite of the memory consumption. The system started to learn successfully within the acceptable limit of time.

Since slight change occurs between adjacent frames, we utilized the frame-skip technique (Bellemare et al., 2013). We took out one frame as the network input by every k + 1 frame, and the same action was repeated over the skipped frames. When k became higher, the training speed became high as well. However, the information the agent got became imprecise as well. In order to achieve the balance of low computing resource consumption and smooth control, we finally choose a frame skip of k = 3 by relying on our experience.

## 3. Experiments

The model was trained using three different tracks, which cover all the track that Stanford used for comparison. An individual set of weights was trained separately for each model because each track has different terrain textures. The rewards were low initially because it is equivalent to a random exploration at the beginning of training and because the driving agent would get stuck somewhere without making any significant progress. After about 1,400 episodes of training, the driving agent finished the race. Under most circumstances, the driving agent did not finish the race in given steps so the reward was positive but not as high as receiving the final big reward. We set this step limit because of a lack of a reset mechanism for dead situations, which was very useful in the early stage of training. In Stanford's report, they created a DNF flag to represent the driving agent getting stuck. In our experiment, the driving agent had learned better policy and displayed better behaviors, proving better robustness of the system. We also visualized the CNN in order to validate the ability of the model.

### 3.1. Experimental Environment

We chose the car racing game to carry out our simulated self-driving experiment. In order to play the car racing game autonomously, we used a third-party OpenAI Gym environment wrapper for the car racing game developed by Bzier^1^. With the assistance of the API, we accessed the game engine directly and ran our code while playing the game frame-by-frame. By means of the API, we can easily get the game information, whether it is screen output intermediate game parameters, such as its location in the small map. Our models proved to efficiently handle the observable gaming environments. To demonstrate the agent's driving status, we include a Youtube link at https://youtu.be/KV-hh8N5x3M.

### 3.2. Rewards Analysis

**Figure 4:**
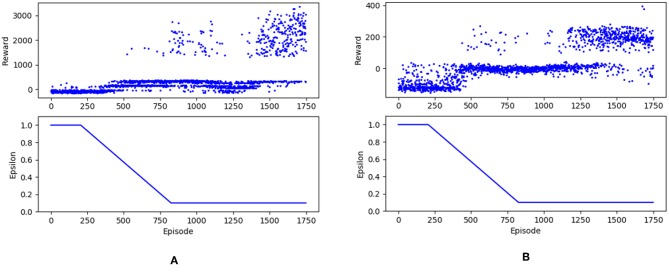
There are reward trends for two different maps. **(A)** corresponded to Farm and **(B)** corresponded to Raceway. After training for 1,750 episodes, we obtained the reward tendency. At about 400 episodes, the stability of the driving agent began to increase. After 1,400 episodes, the reward stabilized at a high level.

For comparison, the model was trained and tested using the same tracks like those used by Stanford in their supervised learning method. Figure 4 shows the rewards trends for two different maps. From the rewards trends we can observe that at about 400 episodes, the stability of the driving agent began to increase. The reward stabilized at a high level after 1400 episodes, where the agent performed a good driving behavior and finished the tracks well. Each track has different terrain textures and difficulty routes. Therefore, an individual set of weights was trained separately for each model. The experiments were carried out on a common configured portable laptop, and all models converged after spending over 80 h each.

### 3.3. CNN Visualization

**Figure 5:**
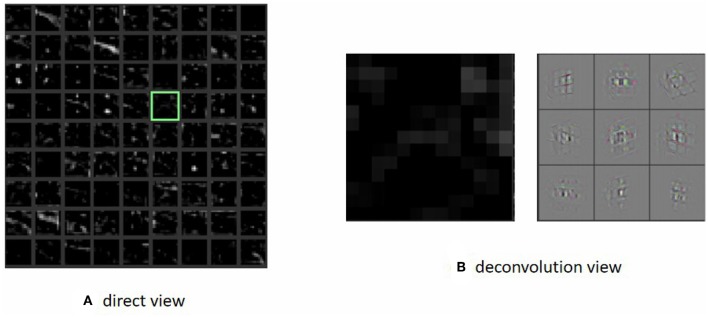
We visualized the first layer and obtain a direct view (left). However, many grids contain unreadable information such as the grid marked with a green frame. Because the first layer's output was quite abstract, we then visualized the last layer using deconvolution and obtained the right picture. It seemed to represent the wall element in the original graph. That means the agent could pay attention to the wall and then take suitable actions. Panel **(A)** shows the direct view visualization of the first layer of CNN. Panel **(B)** shows the visualization of the last layer of CNN using deconvolution.

CNN layers were used to extract abundant information in the scene, and the result of the high-level feature was the critical measurement of the training process. In the traditional supervised learning, the network could be evaluated from many methods such as the validation accuracy and the loss function. However, in the reinforcement learning, we did not have this kind of method to provide a quantitative assessment of the model. Hence, we visualized the output of the first layer and the last layer (see Figure 5), with the aim of ascertaining the quantitative features that are captured by the network. The high-level low-level layer output seemed quite abstract through direct observation. Thus, we visualized the high-level features through deconvolution. Through the visualization procedure, we were sure that the network could capture the important element in the scene.

### 3.4. Result and Discussion

The test results are shown in Table 1. The driving agent was tested on several tracks in line with those of Stanford's experiment such as Farm, Raceway, and Mountain. We evaluated our model based on the time. For each track, we ran 10 races in real-time and calculated the mean race times as the final result. The human results (Ho et al., 2017) and the Stanford results were borrowed from the Stanford report. Human results are obtained by real human participants playing each track two times: The first time is to get used to the track, and the second time is to record the time they finish the game.

**Table 1:** Performance comparison.

**Track**	**Our model**	**Stanford model (Ho et al., 2017)**	**Human**
Farm	98.33	97.46	94.07
Raceway	166	129.09, 1 DNF^*^	125.30
Mountain	213	138.37, 2 DNF^*^	129.50

For each track, we run 10 races in real time and calculate the mean race times as the final result. The Stanford results were borrowed from the Stanford report. Human results are obtained by real human participants playing each track twice: The first time is to get used to the track, and the second time is to record the time they finish the game. DNF

* *signifies that the autopilot got stuck and was unable to finish some number of races*.

The proposed model shows some advantages via a comprehensive comparison in Table 2. Firstly, some of the actions such as braking and going backwards are important in the driving kart scenario. Stanford's paper reported that the agent is unable to handle situations where the agent may have to turn around or drive backward, and thus would lead to getting stuck. Secondly, their AI is more sensitive to positive visual information. If the kart picks the wrong direction, it would likely drive straight since the visual scenes of the true and wrong direction are mostly the same. In other words, their AI does not understand risky situations that are likely to lead to error states during real-time play. Thirdly, the Stanford model was trained in a handcrafted dataset collected from 18,658 training examples across four tracks, three of which were also used for testing. If they want to generalize their model to other tracks, they need to collect new data and annotations, which might be expensive and unfeasible. Intuitively, our proposed model attempts to learn actions by trial and error without a huge amount of labels and handcrafted datasets.

**Table 2:** System comparison between our work and Stanford's supervised model.

**Aspect**	**Details**	**Our agent**	**Stanford's agent**
Skills	Turning left/right, driving forward	✓	✓
	Driving backward	✓	
	Brake	✓	
	Turning around	✓	
Bad situation	Driving reverse		✓
	Getting stuck		✓
Performance comparison	Less time-consuming		✓
	Finishing the tracks	✓	✓
Data-consuming	Handcrafted dataset		✓
	Annotation		✓
	Unlabeled data	✓	
Self-adaptability		✓	
Online learning paradigm		✓	

By analyzing the route the agent runs, we found that the route was not as smooth as that in Stanford's experiment, for which there are two reasons. On one hand, ϵ decayed too fast. As shown in Figure 5, the value of ϵ rapidly decreased to 0.1 while the rewards increased. Then, during the latter phase of training, the agent depended mainly on experiences even though there were still many better state-action sets to explore. On the other hand, the actions was discretized roughly. We used a set of [0; 20; 40; 80] as the choices for speed. Through observation, speeds of 20 and 40 both produce a tiny effect while a speed of 80 would make a radical change. More efforts should put on the selection of numerical value of the joystick parameter. However, compared with the experiment done by the Stanford group, our experiments performed well even if the driving agent deviated from the driveway. We considered the brake and trained the driving agent using a trial-and-error method, which was more in line with the real situation. Hence, the bio-inspired reinforcement learning method in the study was a more suitable approach for the driving agent to make decisions.

## 4. Conclusion

Brain-inspired learning has recently gained additional interest in solving control and decision-making tasks. In this paper, we propose an effective brain-inspired end-to-end learning method with the aim of controlling the simulated self-driving agent. Our modified DRQN model has proven to manage plenty of error states effectively, thus indicating that our trial-and-error method using deep recurrent reinforcement learning could achieve better performance and stability. By using the screen pixels as the only input of the system, our method highly resembles the experience of human beings solving a navigation task from the first-person perspective. This resemblance makes this research inspirational for real-world robotics applications. Hopefully, the proposed brain-inspired learning system will inspire real-world self-driving control solutions.

## Data Availability

No datasets were generated or analyzed for this study.

## Author Contributions

JieC, JinC, RZ, and XH carried out the conception and design of the study, the analysis and interpretation of the data, and drafted and revised the article.

### Conflict of Interest Statement

The authors declare that the research was conducted in the absence of any commercial or financial relationships that could be construed as a potential conflict of interest.

## Footnotes

^1^https://github.com/bzier/gym-mupen64plus
